# Neurophysiological patterns reflecting vulnerability to delirium subtypes: a resting-state EEG and event-related potential study

**DOI:** 10.1093/braincomms/fcae298

**Published:** 2024-09-05

**Authors:** Monique S Boord, Daniel Feuerriegel, Scott W Coussens, Daniel H J Davis, Peter J Psaltis, Marta I Garrido, Alice Bourke, Hannah A D Keage

**Affiliations:** Cognitive Ageing and Impairment Neurosciences Laboratory, Justice and Society, University of South Australia, Adelaide, 5072, South Australia, Australia; College of Education, Psychology and Social Work, Flinders University, Adelaide, 5042, South Australia, Australia; Melbourne School of Psychological Sciences, University of Melbourne, Melbourne, 3052, Victoria, Australia; Cognitive Ageing and Impairment Neurosciences Laboratory, Justice and Society, University of South Australia, Adelaide, 5072, South Australia, Australia; MRC Unit for Lifelong Health and Ageing, UCL, London, WC1E 6BT, UK; Vascular Research Centre, Heart and Vascular Program, Lifelong Health Theme, South Australian Health and Medical Research Institute, Adelaide, 5000, South Australia, Australia; Adelaide Medical School, University of Adelaide, Adelaide, 5005, South Australia, Australia; Department of Cardiology, Royal Adelaide Hospital, Central Adelaide Local Health Network, Adelaide, 5000, South Australia, Australia; Melbourne School of Psychological Sciences, University of Melbourne, Melbourne, 3052, Victoria, Australia; Graeme Clark Institute for Biomedical Engineering, University of Melbourne, Melbourne, 3052, Victoria, Australia; Aged Care, Rehabilitation and Palliative Care (Medical), Northern Adelaide Local Health Network, Adelaide, 5092, South Australia, Australia; Cognitive Ageing and Impairment Neurosciences Laboratory, Justice and Society, University of South Australia, Adelaide, 5072, South Australia, Australia

**Keywords:** delirium, pathophysiology, EEG, event-related potentials

## Abstract

Delirium is a common and acute neurocognitive disorder in older adults associated with increased risk of dementia and death. Understanding the interaction between brain vulnerability and acute stressors is key to delirium pathophysiology, but the neurophysiology of delirium vulnerability is not well defined. This study aimed to identify pre-operative resting-state EEG and event-related potential markers of incident delirium and its subtypes in older adults undergoing elective cardiac procedures. This prospective observational study included 58 older participants (mean age = 75.6 years, SD = 7.1; 46 male/12 female); COVID-19 restrictions limited recruitment. Baseline assessments were conducted in the weeks before elective cardiac procedures and included a 4-min resting-state EEG recording (2-min eyes open and 2-min eyes closed), a 5-min frequency auditory oddball paradigm recording, and cognitive and depression examinations. Periodic peak power, peak frequency and bandwidth measures, and aperiodic offsets and exponents were derived from resting-state EEG data. Event-related potentials were measured as mean component amplitudes (first positive component, first negative component, early third positive component, and mismatch negativity) following standard and deviant auditory stimuli. Incident delirium occurred in 21 participants: 10 hypoactive, 6 mixed, and 5 hyperactive. Incident hyperactive delirium was associated with higher pre-operative eyes open (*P* = 0.045, *d* = 1.0) and closed (*P* = 0.036, *d* = 1.0) aperiodic offsets. Incident mixed delirium was associated with significantly larger pre-operative first positive component amplitudes to deviants (*P* = 0.037, *d* = 1.0) and larger third positive component amplitudes to standards (*P* = 0.025, *d* = 1.0) and deviants (*P* = 0.041, *d* = 0.9). Other statistically non-significant but moderate-to-large effects were observed in relation to all subtypes. We report evidence of neurophysiological markers of delirium risk weeks prior to elective cardiac procedures in older adults. Despite being underpowered due to COVID-19–related recruitment impacts, these findings indicate pre-operative dysfunction in neural excitation/inhibition balance associated with different delirium subtypes and warrant further investigation on a larger scale.

## Teaser

Boord *et al*. report significantly larger pre-operative aperiodic offsets in those who develop hyperactive delirium and significantly larger pre-operative P3 event-related potential amplitudes in those who develop mixed delirium compared to those who did not develop delirium. Findings indicate pre-operative dysfunction in neural excitation/inhibition balance associated with different delirium subtypes.

## Graphical Abstract

**Graphical Abstract:**
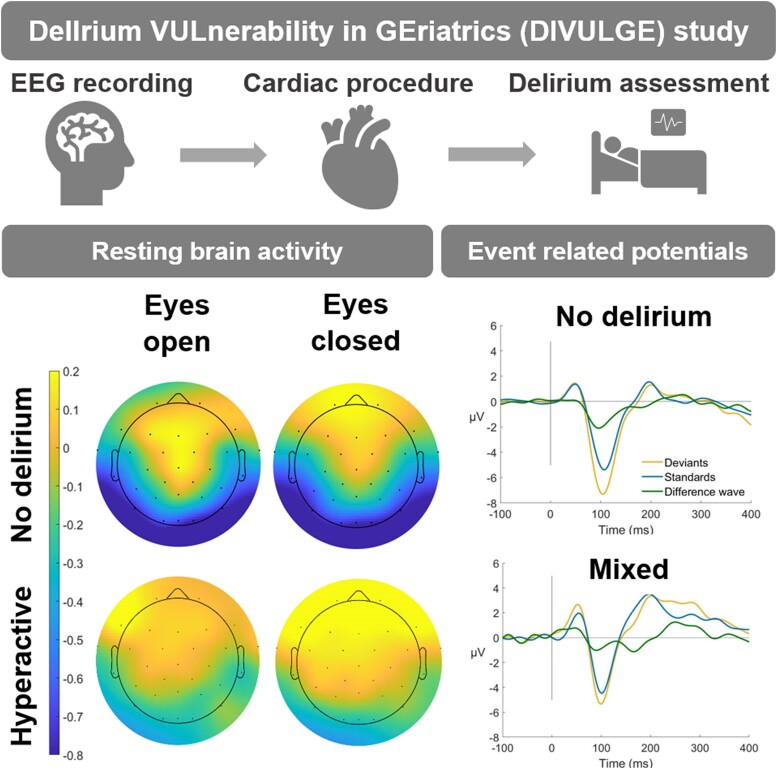

## Introduction

Delirium is a neurocognitive disorder occurring in 25% of older adults undergoing cardiac procedures.^1-3^ The consequences of delirium are severe, including a 9-fold increased dementia risk and a 4-fold increased mortality risk.^4,5^ Delirium subtypes are typically distinguished by patterns of motor activity.^6,7^ Hypoactive delirium is characterized by reduced or absent movement and speech; hyperactive delirium by agitation, hallucinations, and increased motor activity. Mixed delirium has both hypoactive and hyperactive symptoms.^8^ Each subtype requires different management and is associated with different outcomes.^9-12^ However, previous investigations have not typically distinguished subtypes. Although some symptoms overlap, the discriminating hypoarousal and hyperarousal clinical presentations are markedly different. Therefore, different neurobiological processes (e.g. arousal, awareness and attention), or different manifestations of similar processes, are likely at play.^13^

Delirium episodes have been characterized by higher power in the delta and theta bands of the EEG, lower alpha power, and reduced functional connectivity.^14-18^ Current theories describe delirium as a disorder of functional brain disintegration^19-22^ and delirium vulnerability as a state of reduced baseline functional connectivity.^20^ Recently, however, Tanabe *et al.*^23^ reported that those who went on to develop any delirium had higher pre-operative alpha power and functional connectivity. This was the first to suggest a neurophysiological measure before an acute insult (in this case, surgery) could index delirium risk. However, major delirium predisposing factors such as cognitive function and depression^24^ were not controlled for; therefore, it is unclear whether it was predisposing factors or brain vulnerability for delirium driving these effects.

Functional neuroimaging has been successful in revealing patterns of activity specific to subtypes of other psychiatric disorders, including attention deficit hyperactivity disorder^13^ and schizophrenia.^25^ Further, vulnerable or at-risk brain states and patterns are shown in other psychiatric conditions.^26-28^ Event-related potentials (ERPs) measure electrophysiological brain activity on a millisecond-by-millisecond basis.^29^ The auditory oddball paradigm involves a sequence of standard tones interspersed with rare, deviant tones (differing in perceptual aspects e.g. pitch or frequency) presented while EEG is recorded.^30^ ERP components from auditory oddball designs are relevant to delirium as inattention is a crucial diagnostic feature.^31^ The first positive (P1) component is primarily associated with early sensory processing and gating of irrelevant or repetitive stimuli;^32^ the first negative component (N1) is another early component elicited in response to apparent auditory change,^33^ and the early subcomponent of the third positive component (P3a) indexes shifting of attention or orientation to unattended deviant stimuli.^30^ The mismatch negativity (MMN) is the difference wave calculated by subtracting the response to standard stimuli from the deviant stimuli.^29,34^ The MMN is a neural response to rule violations in a sequence of standard stimuli, also conceptualized as a prediction error;^34^ the larger the error, the larger the neural response evoked.^35^ The auditory oddball is an ideal paradigm in delirium research as no behavioural response is required.^36^ Recently, Gjini *et al.*^37^ reported smaller MMN amplitudes during a delirium episode and larger pre-operative MMN amplitudes in those who went on to develop delirium.

For analyses of resting-state EEG recordings, methodological advances have highlighted the need to separate oscillatory and aperiodic components of EEG power spectra.^38^ Resting-state EEG power spectra are typically decomposed into frequency bands linked to cognition, behaviour, and various psychiatric disorders.^38-40^ Resting-state EEG activity consists of rhythmic, repeating patterns (periodic activity) and non-rhythmic signals (aperiodic activity) contributing to frequency band power measures. Oscillations are considered to be narrowband peaks visible in the power spectra,^38^ whereas aperiodic activity can be modelled using functions that predict exponentially decreasing power with increasing frequency. The aperiodic exponent indexes the steepness of the power spectrum (i.e. the rate of power decrease with increasing frequency) and has been theorised to reflect cortical excitation/inhibition ratios e.g. a lower (flatter) exponent reflects a shift away from cortical inhibition and vice versa.^41^ The aperiodic offset indexes the broadband shift across frequencies and is proposed to reflect the extent of neuronal spiking, with lower offsets reflecting slower neural firing rates.^41^ Aperiodic activity has historically been under-studied, having been treated as noise. However, it has been shown to change with age^42^ and is a promising marker of disorders including attention deficit hyperactivity disorder^43,44^ and schizophrenia.^45,46^

Delirium subtype symptom profiles differ, as do their outcomes and care.^9,47^ Therefore, it is crucial to investigate subtypes separately when characterizing neural vulnerability to delirium. Identifying those at risk for a delirium subtype will allow the targeting of known effective interventions in pre-operative care and earlier diagnosis, as well as informing management and prognosis.^48^ Here, we aimed to characterize neural vulnerability to delirium subtypes using ERP component measures from an auditory oddball design and periodic and aperiodic features of EEG power spectra. We hypothesized that those who develop delirium will show (before surgery) EEG slowing (increased delta and theta and decreased alpha power) and lower aperiodic slopes and exponents. An exploratory aim was to determine how these components vary between no delirium and incident delirium subtypes.

## Materials and methods

### Study design and participants

**Figure 1:**
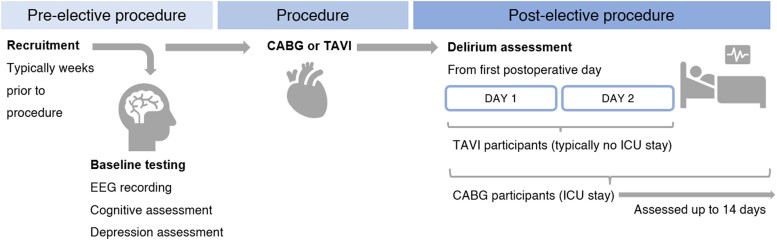
**Study procedure.** EEG, electroencephalography; CABG, coronary artery bypass grafting; TAVI, transcatheter aortic valve implantation; ICU, intensive care unit.

A detailed description of the methods is provided in our published protocol.^49^ Any deviations from the protocol are described below. Data were collected between May 2018 and August 2022 as part of the DelIrium VULnerability in GEriatrics (DIVULGE) study. Participants ≥ 60 years were recruited from two larger trials.^50,51^ Based on power analyses, we aimed to recruit 90 participants with complete recordings.^49^ However, this was not feasible due to COVID-19–related disruptions (The Royal Adelaide Hospital stopped elective procedures multiple times across 2020 and 2022). Ethical approvals were granted from the University of South Australia Ethics Committee (0000034053 and ET00013), as well as human research ethics committees of the Royal Adelaide Hospital (HREC/17/RAH/445 and HREC/17/RAH/391) and the Central Adelaide Local Health Network (R20171020 and R20170916). Data were collected in participants’ homes or the University of South Australia Magill campus in the weeks before their elective procedures. Cognitive assessments, depression assessments, and EEG recordings were performed at baseline. Delirium was assessed once daily from the first post-operative day [up to 14 days in coronary artery bypass grafting (CABG) participants and 2 days in transcatheter aortic valve implantation (TAVI) participants; Fig. 1].

### EEG recording before the elective procedure

We recorded 9 min of 32-channel EEG at a sample rate of 1000 Hz, consisting of 4-min resting-state (2-min eyes open and 2-min eyes closed) and a 5-min passive, frequency alteration, auditory oddball paradigm. Before recording, participants were comfortably seated with their feet placed flat on the floor. During the eyes open resting-state EEG recording, participants were asked to look at a fixation point on the centre of a laptop screen. The auditory oddball paradigm immediately followed, consisting of 300 stimuli with a 150 ms duration and 500 ms interstimulus interval. Standard tones were presented at 600 Hz (23% of stimuli) and deviant tones at 1000 Hz. Participants were directed to watch a silent video on an iPad placed in front of them displaying traffic on a main road in front of the University of South Australia Magill campus. Checks were taken to ensure participants could hear the stimuli before the recording.

### EEG data processing

Data were processed in MATLAB (V.R2019a, MathWorks, USA) using the EEGLAB toolbox (V.v2019.1).^52^ Resting-state EEG Fourier power spectra were parameterized, and periodic and aperiodic components were measured using the Fitting Oscillations and One Over F (FOOOF) toolbox in Python.^38^ Aperiodic exponents were extracted from the 1–30 Hz frequency range (1 and 8 Hz peak width limits, minimum peak height = 0.00, maximum of 6 peaks per power spectrum, and a peak threshold of 2 SD). Aperiodic offsets and exponents were calculated at each electrode. Periodic component peak measures (frequency, power, and bandwidth) were extracted in the delta (1–3 Hz), theta (4–6 Hz), alpha (6–12 Hz), and beta (13–30 Hz) frequency bands.

It was planned in our protocol to extract peaks within delta, theta, alpha, and beta frequency bands;^49^ however, clear peaks were only detected for alpha and beta frequencies. Recent work shows that lack of theta and delta peaks are not unusual in this age range.^53,54^ Further, we had planned to divide electrodes into three regions of interest (anterior, central, and posterior); however, electrodes for alpha and beta frequencies were instead based on topographic maps (Supplementary Fig. 1); this reduced the number of comparisons. For the alpha region of interest (ROI), the following channels were selected: CP1, CP2, P3, Pz, P4, O1, Oz, and O2. The beta ROI consisted of channels F3, Fz, F4, FC5, FC1, FC2, FC6, C3, Cz, C4, CP5, CP1, CP2, CP6, P3, Pz, and P4. Conventional analyses of eyes open and closed relative frequency band power were conducted as described in our protocol.^49^ Results are provided in Supplementary Table 1 (delirium presence versus absence) and Supplementary Table 2 (delirium subtypes versus no delirium groups).

EEG data from oddball sequences were processed in MATLAB and ERPlab v.7.0.0.^55^ Data were re-referenced to TP9 and TP10 electrodes. A high-pass (0.1 Hz), low-pass (40 Hz), and notch (50 Hz) filters were applied, and independent component analysis was performed on the filtered datasets. ICLabel was used to remove bad components at a threshold of 80%. Bad channels were interpolated using the clean data. The data were then low-pass–filtered at 20 Hz. Clean data were segmented from −100 to +400 ms relative to stimulus onset. Epochs with amplitudes >±100 μV were removed. The P1 mean amplitude measurement window was set to 40–60 ms centred around the 50 ms peak; the N1 window was 80–120 ms centred around the 100 ms peak; the P3 window was set at 200–400 ms; and the MMN window was set at 100–200 ms to cover the typical mismatch window.^34^ Mean amplitudes were calculated separately for standard and deviant tones for the P1, N1, and P3 components at a single frontocentral channel (Fz). We initially planned to model 3D spatiotemporal images using a mass-univariate general linear model using statistical parametric mapping.^49^ We could not conduct this analysis due to time constraints and reduced power; ERP component measures are therefore the primary outcomes reported here.

### Cognitive and depression assessment

Cognitive and depression assessments (both primary risk factors for delirium^24^) were undertaken in the larger trials in which this study is nested.^50,51^ Cognition was assessed using the Addenbrooke’s Cognitive Examination III with higher scores indicating better cognitive performance.^56^ Depression was assessed using the Geriatric Depression Scale (GDS) short form with higher scores indicating higher depression severity.^57^

### Delirium assessment post elective procedure

In the intensive care unit (ICU), participants were assessed using the Confusion Assessment Method for the ICU (CAM-ICU).^58^ On surgical wards, participants were assessed using the Memorial Delirium Assessment Scale (MDAS),^59^ which served as an interview to score the short CAM^60^ for delirium presence. Delirium presence was determined by a positive CAM i.e. meeting both features 1 (acute or fluctuating mental status) and 2 (inattention) as well as either feature 3 (altered level of consciousness) or 4 (disorganized thinking). To complement assessment for arousal disturbances, the Observational Scale Level of Arousal was used.^61^ Delirium subtype was determined using a delirium motor subtype checklist.^7^ Participants were characterized as having mixed delirium if hyperactive and hypoactive delirium episodes were present across their hospital admission. If participants were in hospital over the weekend, a validated chart-based review tool was used to ascertain delirium presence and subtype.^62^

### Statistical analyses

Analyses were conducted in Jamovi (version 1.6)^63^ except for SPSS (Version 26, IBM Statistics) for bootstrapping analysis of covariance (ANCOVA). Categorical variables are expressed as count and percentage, and continuous variables are expressed as means ± SD.

Due to COVID-19–related study disruptions to recruitment and reduced sample size, we could not investigate delirium severity and duration as planned^49^ and have focussed on delirium absence versus presence and delirium subtype. The ANCOVA models were used to investigate differences in EEG indices and ERP component amplitudes, controlling for age, cognition, and depression between incident delirium versus no delirium. Our published protocol^49^ detailed age and cognition as covariates. However, we decided to include depression as a further covariate due to its moderate-to-large contribution to risk for delirium.^24^ Effect sizes were calculated using partial eta squared (η_p_^2^) using standard cut-offs: small (η_p_^2^ = 0.01), medium (η_p_^2^ = 0.06), and large (η_p_^2^ = 0.14). Independent samples *t*-tests were used to compare differences in EEG measures between no delirium and delirium subtypes (hypoactive, hyperactive, and mixed). Mann–Whitney U-test or Welch’s *t*-test was used in instances where normality or homogeneity of variance was violated.

Alpha was set at 0.05. We did not correct for multiple comparisons as this is a new area of enquiry, and retaining sensitivity to identify candidate effects (which can be replicated in future studies) in our small sample was deemed important. We therefore focus on effect sizes for our interpretations. Cohen’s *d* was calculated for effect size estimates using standard cut-offs: small (*d* = 0.2), medium (*d* = 0.5), and large (*d* = 0.8).

## Results

### Sample characteristics

Fifty-eight participants were recruited (Table 1; Supplementary Table 3). Five participants were ineligible for inclusion in the ERP analysis due to technical error (no oddball paradigm recording). Hence, 53 participants were included in the ERP analysis. Incident delirium occurred in 21 participants (34%). Hypoactive subtype was present in 10 (48%), followed by mixed delirium in 6 (29%), and hyperactive delirium in 5 (24%) participants. There were no significant differences between those who went on to develop delirium and those who did not, in terms of age, gender, cognition (ACE-III score), and depression (GDS score). There were no significant differences in baseline characteristics between no delirium and delirium subtype (hypoactive, hyperactive, and mixed) groups.

**Table 1:** Participant demographics

	No delirium	Delirium	Hypoactive delirium	Hyperactive delirium	Mixed delirium
n	37	21	10	5	6
Mean age in years (SD)	75.7 (7.4)	75.3 (6.8)	74.5 (7.2)	76.6 (6.7)	75.5 (7.1)
Range of age in years	65–91	65–86	65–86	69–85	67–83
Gender (*N* male/female)	30/7	16/5	8/2	4/1	4/2
Mean ACE-III score (SD)	84.2 (9.8)	84.1 (9.7)	84.9 (13.1)	83.6 (3.1)	83.2 (7.7)
Range of ACE-III scores	54–99	56–96	56–96	79–87	71–92
Mean GDS score (SD)	2.7 (2.2)	3.1 (2.3)	2.3 (2.0)	3.4 (0.5)	4.0 (3.3)

.

### Incident delirium (pooled across subtypes)

**Figure 2:**
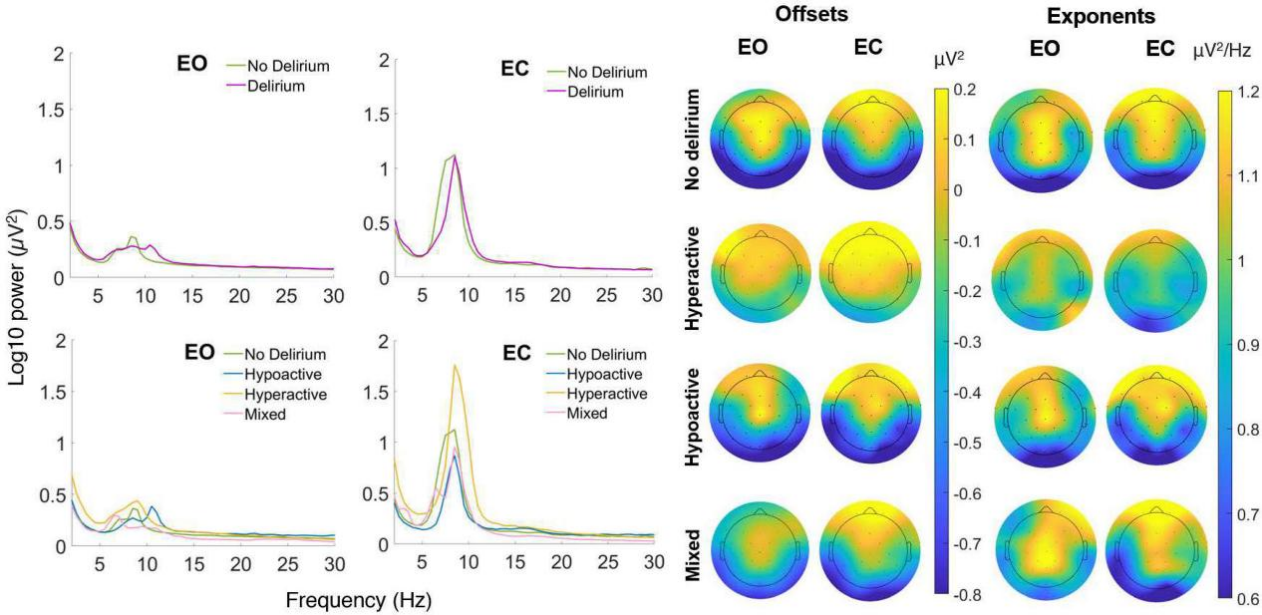
**Group averaged power spectra comparison.** Group averaged power spectra (left) shown for delirium (*n* = 21) versus no delirium (*n* = 37) groups and delirium subtype versus no delirium groups. Group averaged topographic plots (right) show aperiodic offset (μV^2^) and exponent (μV^2^/Hz) during eyes open and closed resting state recording for hypoactive (*n* = 10), hyperactive (*n* = 5), mixed delirium (*n* = 6), and no delirium groups. Independent samples *t*-test show those who developed hyperactive delirium had significantly larger eyes open (*t* = −2.1, *P* = 0.045, *d* = 1.0) and eyes closed (*t* = −2.2, *P* = 0.036, *d* = 1.0) offsets compared to those who did not develop delirium. Remaining comparisons were not significant. EO, eyes open; EC, eyes closed; Hz, hertz; µV², microvolt squared; *t*, test statistic; *P*, *P* value; *d*, Cohen’s *d*.

**Figure 3:**
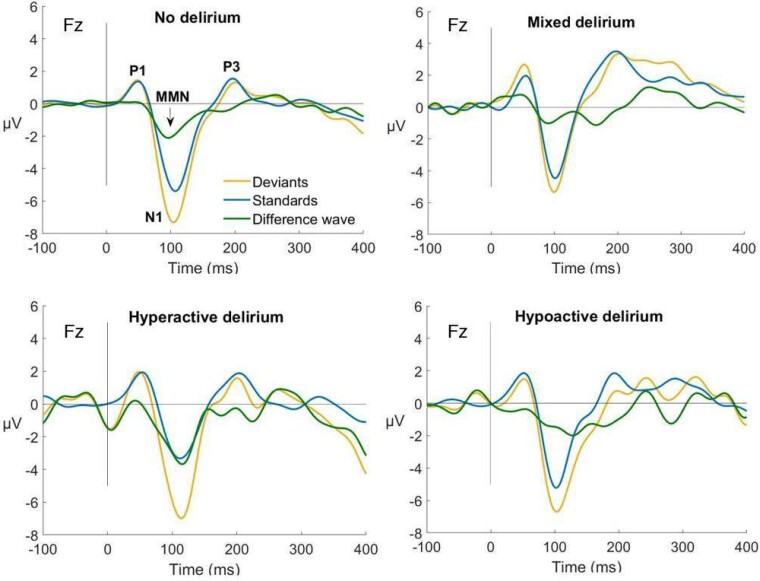
**Event-related potentials comparison.** Event-related potentials for no delirium (*n* = 37), mixed delirium (*n* = 6), hyperactive delirium (*n* = 5), and hypoactive delirium (*n* = 10) groups. Independent samples *t*-test show those who developed mixed delirium had significantly higher P3 amplitudes to standard stimuli (*t* = −2.3, *P* = 0.025, *d* = 1.0), higher P3 amplitudes to deviant stimuli (*t* = −2.1, *P* = 0.041, *d* = 0.9), and higher P1 amplitudes to deviant stimuli (*t* = −2.2, *P* = 0.037, *d* = 1.0) compared to those who did not develop delirium. Remaining comparisons were not significant. P1, first positive component; N1, first negative component; P3, third positive component; ms, milliseconds; µV, microvolts; Fz, frontocentral electrode; MMN, mismatch negativity; *t*, test statistic; *P*, *P* value, *d*, Cohen’s *d*.

**Figure 4:**
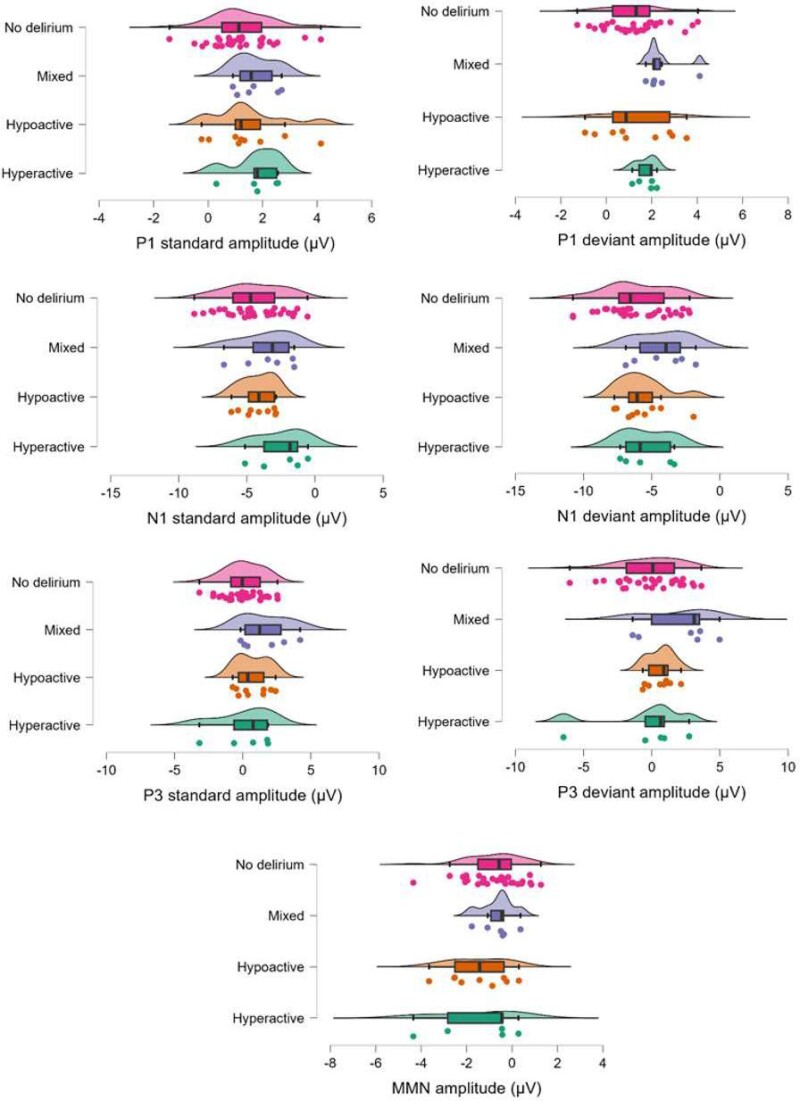
**Raincloud plots displaying individual data of all event-related potential component amplitudes.** No delirium, mixed delirium, hypoactive delirium, and hyperactive delirium groups. P1, first positive component; N1, first negative component; P3, third positive component, µV, microvolts; MMM, mismatch negativity.

Fourier power spectra and scalp maps of offset and exponent values by incident delirium status and subtype are shown in Fig. 2. ERPs for each group are displayed in Fig. 3. Individual data for each ERP component are shown in Fig. 4. Individual data for periodic and aperiodic components in groups with a sample size < 10 are shown in Supplementary Table 8. There were no significant effects of group (delirium presence versus absence) on resting-state EEG or ERP measures. Estimates and effect sizes for comparisons are presented in Supplementary Table 4.

### Incident delirium subtypes

Estimates and effect sizes for eyes open periodic and aperiodic EEG measure comparisons between no delirium and delirium subtype groups are presented in Supplementary Table 5, and eyes closed comparisons in Supplementary Table 6. ERP component amplitude comparisons are presented in Supplementary Table 7.

### No delirium versus hypoactive delirium

No significant differences in any resting-state EEG measures were found (Fig. 2; Supplementary Figs. 2 and 3 and Tables 5, 6, and 7). Moderate effect sizes (not statistically significant) were observed for lower exponents in those who went on to develop hypoactive delirium in both eyes open and closed conditions (Fig. 2). A moderate-to-large effect size was seen for higher MMN amplitude in those who went on to develop hypoactive delirium (Fig. 3).

### No delirium versus hyperactive delirium

Aperiodic offsets for eyes open conditions were larger in those who developed hyperactive delirium (M = −0.1, SD = 0.4 μV^2^), compared to those who did not develop delirium (M = −0.4, SD = 0.3 μV^2^, *t* = −2.1, *P* = 0.045, *d* = 1.0; Figure 2 offsets column). Eyes closed offsets were also larger in those who went on to develop hyperactive delirium (M = 0.0, SD = 0.4 μV^2^) compared to those who did not develop delirium (M = −0.3, SD = 0.3 μV^2^, *t* = −2.2, *P* = 0.036, *d* = 1.0). No significant differences were shown for remaining measures between those who developed hyperactive delirium compared to those who did not develop delirium (Supplementary Figs. 2 and 3 and Tables 5, 6, and 7). Moderate-to-large effect sizes were observed for larger exponents in both eyes open and closed conditions (Fig. 2 exponents column), higher MMN amplitudes, larger P1 standard amplitudes, and lower N1 standard amplitudes in those who went on to develop hyperactive delirium (Fig. 3).

### No delirium versus mixed delirium

In those who developed mixed delirium, P3 amplitude to standard stimuli was significantly higher (M = 1.6, SD = 1.8 μV) compared to those who did not develop delirium (M = 0.1, SD = 1.4 μV, *t* = −2.3, *P* = 0.025, *d* = 1.0; Figure 3). P3 amplitude to deviant stimuli was significantly higher in those who went on to develop mixed delirium (M = 2.1, SD = 2.6 μV) compared to those who did not develop delirium (M = −0.1, SD = 2.3 μV, *t* = −2.1, *P* = 0.041, *d* = 0.9; Figure 3). P1 amplitudes to deviant stimuli were significantly higher in those who went on to develop mixed delirium (M = 2.4, SD = 0.9 μV) compared to those who did not develop delirium (M = 1.2, SD = 1.3 μV, *t* = −2.2, *P* = 0.037, *d* = 1.0; Fig. 3). No significant differences were observed for other measures (Supplementary Tables 5, 6, and 7). Moderate-to-large effect sizes were observed for larger exponents in both eyes open and closed conditions (Fig. 2), higher P1 standard and deviant amplitudes, and lower N1 deviant amplitudes in those who went on to develop mixed delirium (Fig. 3).

## Discussion

We report associations between pre-operative resting-state EEG measures and incident hyperactive delirium, as well as pre-operative ERP component measures and incident mixed delirium. These neurophysiological markers of delirium vulnerability in older patients were captured in the weeks preceding a delirium episode. Those who developed mixed delirium displayed markers of sensory gating deficits and hyperarousal as indexed by significantly increased pre-operative P1 and P3 amplitudes during a (passive) auditory oddball paradigm. Those who developed hyperactive delirium displayed markers of increased neural firing rates^41^ as indexed by significantly higher pre-operative resting-state offsets. Non-significant but moderate-to-large effect sizes were shown for several EEG and ERP measures across subtypes. We provide tentative evidence that delirium vulnerability can be predicted using pre-operative measures of brain activity and that activity patterns vary across delirium subtypes.

Disturbances in arousal networks may be crucial to understanding delirium vulnerability. The ascending reticular activating system contains various nuclei from the brainstem to the cortex, and neuromodulation from arousal neurons shape regional processing which determine levels of excitability.^64^ Noradrenergic, cholinergic, and dopaminergic neurons are some of the key cell populations of the ascending arousal system; in which delicate changes in concentrations can alter dynamically coordinated cortical activity.^64,65^ Neurotransmitter dysfunction has long been implicated in delirium pathophysiology with some reference to delirium subtypes e.g. serotonin and hypoactive delirium, norepinephrine and hyperactive delirium, and dopamine and mixed delirium.^66,67^ Subcortical arousal systems were implicated in delirium decades ago by Ross *et al*.^68^ and Trzepacz *et al*.,^69^ and altered arousal has been shown to predict delirium.^70^ The ascending reticular arousal system initiates and maintains wakefulness and arousal and shapes cortical dynamics in the brain, particularly those underlying cognition and attention.^64,71,72^ Attention and arousal are inextricably linked and reflected in ERP morphology e.g. greater attention producing greater P3 amplitudes.^73^ Our findings are consistent with the possibility that disturbed cognitive and attentional processes being due to interactions between the ascending arousal system and cortical networks.^71^

Our ERP findings may be indexing abnormal thalamo-cortical dynamics in those vulnerable to developing mixed delirium. Involvement of the ascending arousal system has been implicated previously in delirium (i.e. regardless of subtype) via functional neuroimaging.^74-76^ Reduced functional connectivity between intralaminar thalamic and caudate nuclei and subcortical regions was seen during delirium. However, this reversed upon delirium resolution.^74^ Another study reported decreased arousal network activity and imbalanced cortico-subcortical hemispheric connectivity in right-hemisphere stroke survivors with delirium.^75^ Pre-operatively, increased thalamic mean diffusivity has been reported in those who went on to develop delirium.^76^ One study reported structural dysconnectivity in fronto-thalamo-cerebellar networks in those at risk of delirium, suggesting impairment of key arousal and attentional networks.^77^ Reticulo-thalamo-cortical connectivity, in particular, is key in transitioning from one arousal state to another.^72^ The locus coeruleus, a main source of noradrenaline, is an important component of the ascending arousal system as its neurons control attention and alertness.^64,78^ Phasic (short bursts) locus coeruleus activity has been shown to generate the P3 component.^79^ Mixed delirium is associated with worse outcomes in terms of longer duration, hospital stay, higher mortality, and receiving more pharmacological interventions.^47^ Mixed delirium attracts greater symptom burden^80^ and is characterized by fluctuations or switching between arousal states (hyperactive and hypoactive), likely driven by ascending arousal systems.^64,81^ There is debate as to whether mixed delirium is a homogeneous pathological entity or a mix of hyperactive and hypoactive episodes;^47^ our finding of larger pre-operative P3 amplitudes in those who develop incident mixed delirium provide support for the former.

ERPs in the context of delirium are under-studied. Recent work reported significantly smaller MMN amplitudes during post-operative delirium compared to those without delirium and larger pre-operative MMN amplitudes in those who went on to develop incident delirium, compared to (i) their post-operative MMN amplitudes during a delirium episode and (ii) to those who did not go on to develop delirium.^37^ We observed non-significant but moderate effects for increased pre-operative MMN amplitude in those who developed hyperactive delirium and decreased pre-operative MMN amplitude in those who developed hypoactive delirium. Gjini *et al.*^37^ did not investigate subtypes, so, it’s unclear whether our findings align (e.g. they may have had a high proportion of hyperactive delirium) or not. They also did not investigate P3 amplitudes statistically however visually they appear larger pre-operatively to standard tones in those who went on to develop delirium, which aligns with our finding of significantly larger P3 amplitudes in those who developed mixed delirium.

Our observation of larger pre-operative offsets in those who develop hyperactive delirium could tentatively be interpreted as increased spiking rate of cortical neurons.^41^ Hyperactive delirium occurs less frequently compared to hypoactive or mixed delirium and is associated with distressing symptoms including agitation and hallucinations and higher pharmacological intervention.^47^ Arousal is linked with aperiodic activity i.e. low arousal reflecting reduced excitation and increased offsets and exponents and vice versa;^82,83^ however, larger offsets have been reported in disorders of hyperarousal i.e. non-medicated children with attention deficit hyperactivity disorder, compared to healthy age-matched controls.^84^ The current study measured offsets pre-operatively before the onset of delirium, and it is currently unknown how aperiodic activity differs between a vulnerability state and during the delirium episode.

We found (not statistically significant) moderate effect sizes for differences in aperiodic exponent in those who developed hypoactive delirium i.e. smaller aperiodic exponent (*d* = 0.5). In contrast, higher pre-operative exponents were shown for mixed (*d* = 0.7) and hyperactive delirium (*d* = 0.5). This appears to reflect differing extents or patterns of the same neurobiological process generating delirium subtypes.^85^ It has been suggested that increased neural noise results in desynchronized neural activity, reflected in a flatter exponent driven by an increased excitation/inhibition ratio.^42^ Excitation/inhibition balance is crucial for neural homeostasis and cortical functioning,^86^ and our findings show this balance may be impaired in those vulnerable to developing delirium.

We provide support for investigating delirium subtypes as separate entities, as significant effects were only shown in subtype analyses compared to those comparing delirium presence versus absence.^6^ Our findings showed that when looking at ‘any delirium’, delirium subtype effects are nulled, as they were often in different directions as compared to ‘no delirium’.^87^ Future work needs to replicate these findings in a larger more powered sample pre-, during, and post-delirium. Further, the reliability of pre-operative EEG and ERP measures in differentiating risk for delirium subtypes should be measured over time, within participants, and across samples. Currently there is no universal tool to predict delirium;^88^ its risk factors are heterogeneous^89^ and have recently shown to differ relative to subtype.^6^ Our findings suggest evidence for patterns of brain activity predisposing individuals to delirium subtypes; incorporating these activity profiles may improve delirium prediction and improve sensitivity.^6^ These findings develop a case for subtypes and arousal dynamics to be considered in future investigations. Future work with more data collected before, during, and after delirium should investigate arousal dynamics with advanced methods e.g. investigating structural connectivity strength between noradrenergic and cholinergic arousal system hubs and functional magnetic resonance imaging (fMRI) to assess functional brain networks.^90^

Findings should be interpreted cautiously. Due to our design, we were unable to balance sample size in the delirium subtype groups. In order to retain sensitivity to identifying promising neural candidates (for future replication), we did not control for multiple comparisons and acknowledge the risk for type I errors is increased. Disruptions to study recruitment due to COVID-19 were substantial (multiple state-wide lockdowns between 2020 and 2022). As a result, the study is underpowered, and we have relied on effect sizes for interpretation. We attempted to extract peak values from delta, theta, alpha, and beta frequencies; however, the FOOOF algorithm only reliably identified and extracted peaks from alpha and theta bands. Previous studies utilizing comparable age groups were unable to identify delta and theta peaks using the FOOOF algorithm (albeit slightly different settings).^53,54^ Utilizing task-based measures e.g. during working memory tasks in future may evoke measurable theta activity.^42,91,92^ Under the FOOOF framework, if power is present, it should be apparent through a Gaussian peak rising above the aperiodic activity;^39^ if no peak is detected, this may be consistent with no periodic activity at the given frequency and may likely reflect aperiodic activity.^38^ This does not imply that there is no periodic power at these frequencies; however, it does reinforce the importance of parameterising periodic and aperiodic components of EEG power in future work due to potential conflation of periodic with aperiodic activity.^38^ We utilized a chart review method in the instance participants were in hospital over the weekend which was potentially limited by minimal charting or recognition of delirium. In the current study, delirium was rarely explicitly documented in medical records. However, in some instances, inferences could be made through descriptions of symptoms. Chart reviews did however improve over time due to study staff presence increasing recognition of delirium. Lastly, though part of our pre-planned analyses, categorization of delirium subtypes is being challenged. Recent work encourages moving beyond categorizing delirium subtype purely based on motor activity and considering specific neuropsychiatric symptoms and pathophysiological disturbance.^93^ A recent subtyping initiative has put forward that analysing ‘any delirium’ groups may hinder understanding of delirium pathophysiology and that research should account for underlying biology to improve specificity in delirium.^94^ Our findings provide evidence for neurobiological differences in delirium subtype vulnerability. The subtyping used in this study is widely accepted, though moving forward there may be better and more specific subtyping approaches to consider.

## Conclusion

Neurophysiological markers of arousal dynamics are altered in older adults before developing different delirium subtypes. This study provides novel insight into neural pathophysiology of delirium, extending previous work by incorporating more robust measures and investigating delirium vulnerability at the subtype level. Our findings support theoretically and empirically considering delirium subtypes as separate entities rather than grouping them together, particularly mixed delirium. Further, these findings provide neurophysiological targets to address in future work regarding delirium risk prediction and prevention. By objectively characterizing profiles of neural vulnerability to delirium and its subtypes, risk may be indexed before elective surgery and planning for prevention and management can be achieved when interventions work best. EEG is a promising avenue for delirium prediction and may improve early planning, care management, and early implementation of preventative measures in the pre-operative period.^9^

## Supplementary Material

fcae298_Supplementary_Data

## Data Availability

Data are available upon request. Code used during the study is available in an Open Science Framework (OSF) repository at https://osf.io/nxsd7/.
