# Supplementary material for: Neurophysiological patterns reflecting vulnerability to delirium subtypes: a resting-state EEG and event-related potential study
Source: Brain Commun. 2024 Sep 5;6(5):fcae298. doi: 10.1093/braincomms/fcae298 (PMC11389613; doi:10.1093/braincomms/fcae298)
Supplement: fcae298_Supplementary_Data [file fcae298_supplementary_data.docx]

**Supplementary material**

**Supplementary Figure 1.** Topographic maps of peak power averaged across participants (n=58) used for selecting regions of interest, showing power focused at parietal and occipital electrodes for alpha (left), and more broadly spread power for beta (right).


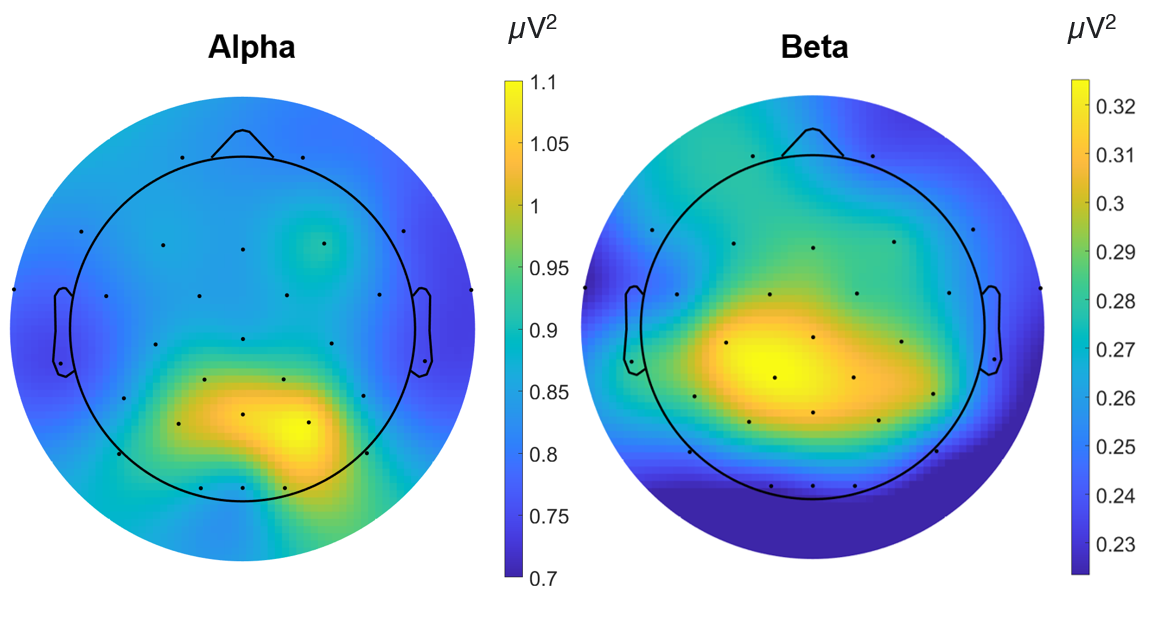


**Resting-state EEG Relative Frequency Band Power Analyses**

| **Supplementary Table 1**. ANCOVA estimates of effect of delirium presence versus absence on periodic and aperiodic EEG measures and ERP measures. | |
| --- | --- |
|  | **Estimates** |
| **Eyes open relative power** | |
| Delta | F(1,53)=0.15, p=.704, η_p_^2^=0.0, age F(1,53)=6.4, p=.014*, η^2^=0.11 (small) |
| Theta | F(1,53)=1.2, p=.274, η_p_^2^=0.02, no significant covariates |
| Alpha | F(1,53)=0.07, p=.797, η_p_^2^=0.0, no significant covariates |
| Beta | F(1,53)=0.05, p=.828, η_p_^2^=0.0, no significant covariates |
| **Eyes closed relative power** | |
| Delta | F(1,53)=0.11, p=.737, η_p_^2^=0.0, cognition F(1,53)=11.3, p=.001*, η_p_^2^=0.18 (large) |
| Theta | F(1,53)=0.1, p=.919, η_p_^2^=0.0, cognition F(1,53)=6.2, p=.016*, η_p_^2^=0.11 (medium to large) |
| Alpha | F(1,53)=0.15, p=.704, η_p_^2^=0.0, no significant covariates |
| Beta | F(1,53)=0.15, p=.701, η_p_^2^=0.0, no significant covariates |

| **Supplementary Table 2.** Eyes open and closed relative power in those who developed delirium subtypes compared to those who did not develop delirium | | | | |
| --- | --- | --- | --- | --- |
|  | **No delirium** | **Hypoactive delirium** | **Hyperactive delirium** | **Mixed delirium** |
| **Eyes open** | | | | |
| Delta | M=0.23 (SD=0.1) | M=0.20 (SD=0.1), *t*=0.8, p=.455, d=0.3 (small), no significant effect. | M=0.22 (SD=0.1), *t*=0.0, p=.982, d=0.0 (small), no significant effect. | M=0.22 (SD=0.1), *t*=-0.9, p=.349, d=0.4 (small to moderate), no significant effect. |
| Theta | M=0.14 (SD=0.1) | M=0.17 (SD=0.1), *U*=163, p=.576, r=0.1 (small), no significant effect. | M=0.19 (SD=0.1), *U*=70.5, p=.404, r=0.2 (small), no significant effect. | M=0.13 (SD=0.1), *U*=89.5, p=.462, r=0.2 (small), no significant effect. |
| Alpha | M=0.3 (SD=0.1) | M=0.3 (SD=0.1), *t*=0.1, p=.882, d=0.1 (small), no significant effect. | M=0.3 (SD=0.1), *t*=0.3, p=.744, d=0.2 (small), no significant effect. | M=0.2 (SD=0.1), *t*=1.5, p=.153, d=0.6 (moderate), no significant effect. |
| Beta | M=0.19 (SD=0.1) | M=0.20 (SD=0.1), *t*=-0.3, p=.769, d=0.1 (small) no significant effect. | M=0.17 (SD=0.1), *U*=67, p=.332, r=0.3 (small), no significant effect. | M=0.18 (SD=0.1), U=109, p=.958, r=0.0 (small), no significant effect. |
| **Eyes closed** | | | | |
| Delta | M=0.21 (SD=0.1) | M=0.2 (SD=0.1), *U*=183, p=.959, r=0.0 (small), no significant effect. | M=0.19 (SD=0.1), *U*=80, p=.641, r=0.1 (small), no significant effect. | M=0.25 (SD=0.1), t=-1.2, p=.250, d=0.5 (moderate), no significant effect. |
| Theta | M=0.21 (SD=0.1) | M=0.2 (SD=0.1), *U*=171, p=.726, r=0.1 (small), no significant effect. | M=0.27 (SD=0.2), *U*=81, p=.678, d=0.1 (small), no significant effect. | M=0.17 (SD=0.1), U=78.5, p=.262, r=0.3 (small), no significant effect. |
| Alpha | M=0.22 (SD=0.1) | M=0.25 (SD=0.1), *t*=-0.9, p=.391, d=0.3 (small), no significant effect. | M=0.19 (SD=0.1), *t*=0.7, p=.483, d=0.3 (small), no significant effect. | M=0.17 (SD=0.1), t=1.3, p=.209, d=0.6 (moderate), no significant effect. |
| Beta | M=0.14 (SD=0.1) | M=0.17 (SD=0.1), *t*=-1.3, p=.194, d=0.5 (moderate), no significant effect. | M=0.12 (SD=0.1), *U*=63.5, p=.268, r=0.3 (small), no significant effect. | M=0.15 (SD=0.1), t=-0.3, p=.733, d=0.2 (small), no significant effect. |
| Note. *t*=independent samples t-test statistic; *U*=Mann-Whitney U test statistic; *r*=rank biserial correlation. | | | | |

| **Supplementary Table 3.**  Participant demographics for ERP analysis | | | | | |
| --- | --- | --- | --- | --- | --- |
|  | No delirium | Delirium | Hypoactive delirium | Hyperactive delirium | Mixed delirium |
| n | 33 | 20 | 9 | 5 | 6 |
| Mean age in years (SD) | 76.0 (7.5) | 75.0 (6.8) | 73.9 (7.3) | 76.6 (6.7) | 75.5 (7.2) |
| Range of age in years | 65-91 | 65-86 | 65-86 | 69-85 | 67-83 |
| Gender (N male/female) | 28/5 | 16/4 | 8/1 | 4/1 | 4/2 |
| Mean ACE-III score (SD) | 85.9 (7.8) | 83.8 (9.9) | 84.3 (13.8) | 83.6 (3.1) | 83.2 (7.6) |
| Range of ACE-III scores | 71-99 | 56-96 | 56-96 | 79-87 | 71-92 |
| Mean GDS score (SD) | 2.5 (2.1) | 3.2 (2.1) | 2.4 (2.1) | 3.4 (0.5) | 4 (3.3) |

There were no significant differences between those who went on to develop delirium and those who did not, in terms of baseline characteristics of age, gender, cognition (ACE-III score), and depression (GDS score). Additionally, no significant differences in baseline characteristics were shown between delirium subtypes and no delirium groups.

| **Supplementary Table 4**. ANCOVA estimates of effect of delirium presence versus absence on periodic and aperiodic EEG measures and ERP measures. | |
| --- | --- |
|  | **Estimates** |
| **Eyes open periodic and aperiodic measures** | |
| Alpha BW (μV^2^) | F(1,49)=0.05, p=.833, η_p_^2^=0.01, no significant covariates |
| Alpha PP (μV^2^) | F(1,49)=0.5, p=.494, η_p_^2^=0.01, no significant covariates |
| Alpha PF (μV^2^) | F(1,49)=0.2, p=.674, η_p_^2^=0.00, no significant covariates |
| Beta BW (μV^2^) | F(1,53)=0.5, p=.472, η_p_^2^=0.01, no significant covariates |
| Beta PP (μV^2^) | F(1,53)=0.1, p=.748, η_p_^2^=0.00, ACE-III scores F(1,53)=6.0, p=.017*, η_p_^2^=0.10 (medium to large) |
| Beta PF (μV^2^) | F(1,53)=0.4, p=.518, η_p_^2^=0.00, no significant covariates |
| Offset (μV^2^) | F(1,53)=0.8, p=.371, η_p_^2^=0.02, no significant covariates |
| Exponent (μV^2^/Hz) | F(1,53)=0.1, p=.795, η_p_^2^=0.00, no significant covariates |
| **Eyes closed periodic and aperiodic measures** | |
| Alpha BW (μV^2^) | F(1,53)=1.2, p=.281, η_p_^2^=0.02, no significant covariates |
| Alpha PP (μV^2^) | F(1,53)=1.0, p=.312, η_p_^2^=0.02, ACE-III scores F(1,53)=6.5, p=.014*, η 2=0.12 (medium to large) |
| Alpha PF (μV^2^) | F(1,53)=0.5, p=.477, η_p_^2^=0.01, ACE-III scores F(1,53)=5.7, p=.020*, η2=0.10 (medium to large) |
| Beta BW (μV^2^) | F(1,53)=0.8, p=.376, η_p_^2^=0.02, no significant covariates |
| Beta PP (μV^2^) | F(1,53)=0.0, p=.970, η_p_^2^=0.00, no significant covariates |
| Beta PF (μV^2^) | F(1,53)=0.2, p=.687, η_p_^2^=0.00, no significant covariates |
| Offset (μV^2^) | F(1,53)=0.2, p=.628, η_p_^2^=0.00, no significant covariates |
| Exponent (μV^2^/Hz) | F(1,53)=0.0, p=.862, η_p_^2^=0.00, no significant covariates |
| **ERPs** | |
| P1 Standard (μV) | F(1,48)=3.0, p=.092, η_p_^2^=0.06, no significant covariates |
| P1 Deviant (μV) | F(1,48)=2.2, p=.141, η_p_^2^=0.05, no significant covariates |
| N1 Standard (μV) | F(1,48)=1.5, p=.226, η_p_^2^=0.03, no significant covariates |
| N1 Deviant (μV) | F(1,48)=1.1, p=.300, η_p_^2^=0.02, age F(1,48)=6.9, p=.012, η_p_^2^=0.13 (medium to large) |
| P3 Standard (μV) | F(1,48)=2.5, p=.119, η_p_^2^=0.05, no significant covariates |
| P3 Deviant (μV) | F(1,48)=0.9, p=.344 η_p_^2^=0.02, no significant covariates |
| MMN (μV) | F(1,48)=2.2, p=.148, η_p_^2^=0.04, age F(1,48)=5.0, p=.029, η_p_^2^= 0.1 (medium to large) |
| Note. BW= bandwidth; PP= peak power; PF= peak frequency; μV= microvolts; μV^2^= microvolts squared; Hz= hertz. | |

| **Supplementary Table 5.** Eyes open periodic and aperiodic components in those who developed delirium subtypes compared to those who did not develop delirium | | | | |
| --- | --- | --- | --- | --- |
|  | **No delirium** | **Hypoactive delirium** | **Hyperactive delirium** | **Mixed delirium** |
| Alpha BW (μV^2^) | M=2.3 (SD=0.7) | M=2.3 (SD=0.5), *t*=-0.2, *p*=.881, *d*=0.1 (small), No significant effect | M=2.3 (SD=0.8), *t*=-0.0, *p*=.996, *d*=0.0 (small), No significant effect | M=2.1 (SD=0.8), *t*=0.4, *p*=.709, *d*=0.2 (small), No significant effect |
| Alpha PP (μV^2^) | M=0.4 (SD=0.3) | M=0.4 (SD=0.3), *U*=99, *p*=.392, *r*=0.2 (small), No significant effect | M=0.6 (SD=0.4), *U*=78.0, *p*=.721, *r*=0.1 (small), No significant effect | M=0.5 (SD=0.3), *U*=102.0, *p*=.847, *r*=0.0 (small), No significant effect |
| Alpha PF (μV^2^) | M=9.3 (SD=1.2) | M=9.7 (SD=1.2), *t*=-0.8, *p*=.441, *d*=0.3 (small), No significant effect | M=9.3 (SD=0.9), *t*=-0.0, *p*=.924, *d*=0.0 (small), No significant effect | M=9.3 (SD=1.2), *t*=0.1, *p*=.943, *d*=0.0 (small), No significant effect |
| Beta BW (μV^2^) | M=4.0 (SD=1.1) | M=4.0 (SD=1.4), *U*=165, *p*=.884, *r*=0.0 (small), No significant effect | M=4.4 (SD=1.4), *U*=73.0, *p*=.472, *r*=0.2 (small), No significant effect | M=4.2 (SD=1.2), *U*=91.0, *p*=.451, *r*=0.2 (small), No significant effect |
| Beta PP (μV^2^) | M=0.3 (SD=0.1) | M=0.3 (SD=0.1), *U*=161, *p*=.801, *r*=0.1 (small), No significant effect | M=0.3 (SD=0.1), *U*=75.0, *p*=.520, *r*=0.2 (small), No significant effect | M=0.3 (SD=0.1), *t*=104.0, *p*=.751, *r*=0.1 (small), No significant effect |
| Beta PF (μV^2^) | M=18.5 (SD=2.6) | M=18.6 (SD=3.1), *t*=-0.2, *p*=.880, *d*=0.1 (small), No significant effect | M=19.1 (SD=4.6), *W*=-0.3, *p*=.797, *d*=0.2 (small), No significant effect | M=19.6 (SD=3.0), *t*=-0.9, *p*=.358, *d*=0.4 (small to moderate), No significant effect |
| Offset (μV^2^) | M=-0.4 (SD=0.3) | M=-0.4 (SD=0.3), *t*=0.5, *p*=.638, *d*=0.2 (small), No significant effect | M=-0.1 (SD=0.4), *t*=-2.1, *p*=.045*, *d*=1.0 (large), Hyperactive significantly higher | M=-0.3 (SD=0.2), *t*=-0.8, *p*=.431, *d*=-0.3 (small), No significant effect |
| Exponent (μV^2^/Hz) | M=0.7 (SD=0.3) | M=0.5 (SD=0.3), *t*=1.3, *p*=.189, *d*=0.5 (moderate), No significant effect | M=0.8 (SD=0.4), *t*=-1.0, *p*=.309, *d*=0.5 (moderate), No significant effect | M=0.9 (SD=0.3), *t*=-1.5, *p*=.146, *d*=0.7 (moderate to large), No significant effect |
| Note. *t*=independent samples t-test statistic; *U*=Mann-Whitney U test statistic; *r*=rank biserial correlation; BW= bandwidth; PP=peak power; PF=peak frequency; μV^2^= microvolts squared; Hz= hertz. | | | | |

| **Supplementary Table 6.** Eyes closed periodic and aperiodic components in those who developed delirium subtypes compared to those who did not develop delirium | | | | |
| --- | --- | --- | --- | --- |
|  | **No delirium** | **Hypoactive delirium** | **Hyperactive delirium** | **Mixed delirium** |
| Alpha BW (μV^2^) | M=2.1 (SD=0.5) | M=2.2 (SD=0.8), *t*=-0.5, *p*=.629, *d*=0.2 (small), No significant effect | M=2.3 (SD=0.7), *t*=-0.9, *p*=.361, *d*=0.4 (small to moderate), No significant effect | M=2.3 (SD=0.2), *W*=-2.1, *p*=.054, *d*=0.6 (moderate), No significant effect |
| Alpha PP (μV^2^) | M=1.0 (SD=0.4) | M=0.7 (SD=0.5), *t*=1.9, *p*=.070, *d*=0.7 (moderate to large), No significant effect | M=1.1 (SD=0.5), *t*=-0.8, *p*=.444, *d*=0.4 (small to moderate), No significant effect | M=0.9 (SD=0.4), *t*=0.3, *p*=.784, *d*=0.1 (small), No significant effect |
| Alpha PF (μV^2^) | M=9.0 (SD=1.1) | M=9.4 (SD=1.3), *t*=-1.1, *p*=.251, *d*=0.4 (small to moderate), No significant effect | M=9.1 (SD=0.7), *t*=-0.3, *p*=.732, *d*=0.2 (small), No significant effect | M=8.8 (SD=1.7), *t*=0.1, *p*=.904, *d*=0.1 (small), No significant effect |
| Beta BW (μV^2^) | M=4.0 (SD=1.4) | M=5.0 (SD=1.5), *t*=-1.3, *p*=.190, *d*=0.5 (moderate), No significant effect | M=4.8 (SD=1.1), *t*=-1.2, *p*=.248, *d*=0.6 (moderate), No significant effect | M=3.7 (SD=1.8), *U*=103.0, *p*=.726, *r*=0.1 (small), No significant effect |
| Beta PP (μV^2^) | M=0.3 (SD=0.1) | M=0.3 (SD=0.1), *t*=0.2, *p*=.815, *d*=0.1 (small), No significant effect | M=0.3 (SD=0.1), *t*=-0.3, *p*=.776, *d*=0.1 (small), No significant effect | M=0.3 (SD=0.1), *t*=0.0, *p*=.995, *d*=0.0 (small), No significant effect |
| Beta PF (μV^2^) | M=17.6 (SD=2.8) | M=17.0 (SD=3.1), *U*=131.0, *p*=.291, *r*=0.2 (small), No significant effect | M=16.7 (SD=2.9), *U*=69.0, *p*=.383, *r*=0.3 (small), No significant effect | M=17.9 (SD=2.2), *U*=97.0, *p*=.582, *d*=0.1 (small), No significant effect |
| Offset (μV^2^) | M=-0.3 (SD=0.3) | M=-0.4 (SD=0.3), *t*=1.1, *p*=.266, *d*=0.4 (small), No significant effect | M=0.0 (SD=0.4), *t*=-2.2, *p*=.036*, *d*=1.0 (large), Hyperactive significantly higher | M=-0.2 (SD=0.3), *t*=-0.7, *p*=.509, *d*=0.3 (small), No significant effect |
| Exponent (μV^2^/Hz) | M=0.8 (SD=0.3) | M=0.6 (SD=0.3), *t*=1.5, *p*=.139, *d*=0.5 (moderate), No significant effect | M=0.9 (SD=0.4), *t*=-1.1, *p*=.300, *d*=0.5 (moderate), No significant effect | M=1.0 (SD=0.3), *t*=-1.6, *p*=.125, *d*=0.7 (moderate to large), No significant effect |
| Note. *t*=independent samples t-test statistic; *U*=Mann-Whitney U test statistic; *r*=rank biserial correlation; BW= bandwidth; PP=peak power; PF=peak frequency; μV^2^= microvolts squared; Hz= hertz. | | | | |

| **Supplementary Table 7.** ERP amplitudes to standard stimuli in those who developed delirium subtypes compared to those who did not develop delirium | | | | |
| --- | --- | --- | --- | --- |
|  | **No delirium** | **Hypoactive delirium** | **Hyperactive delirium** | **Mixed delirium** |
| P1 standard amplitude (µV) | M=1.2 (SD=1.1) | M=1.5 (SD=1.4), *t*=-0.6, *p*= 0.554, *d*=0.2, (small), No significant effect | M=1.8 (SD=0.9), *t*=-1.1, *p*=.228, *d*=0.5 (moderate), no significant effect | M=1.7 (SD=0.8), *t*=-1.1, *p*=.290, *d*=0.5 (moderate), no significant effect |
| P1 deviant amplitude (µV) | M=1.2 (SD=1.3), | M=1.2 (SD=1.3), *t*=-0.1, *p*= .901, *d*=0.0, (small), no significant effect | M=1.8 (SD=0.5), *t*=-0.9, *p*=.387, *d*=0.4 (small), no significant effect | M=2.4 (SD=0.9), *t*=-2.2, *p*=.037*, *d*=1.0 (large), Mixed delirium significantly higher |
| N1 standard amplitude (µV) | M=-4.5 (SD=2.2) | M=-4.2 (SD=1.2), *t*=-0.4, *p*= .722, *d*=0.1, (small), no significant effect | M=-2.5 (SD=1.9), *t*=-1.9, *p*=.064, *d*=0.9 (large), no significant effect | M=-3.5 (SD=2.0), *t*=-1.0, *p*=.318, *d*=0.4 (small), no significant effect |
| N1 deviant amplitude (µV) | M=-6.0 (SD=2.4), | M=-5.7 (SD=1.8), *t*=-0.5, *p*= .649, *d*=0.2, (small), no significant effect | M=-5.4 (SD=1.8), *t*=-0.6, *p*=.547, *d*=0.3 (small), no significant effect | M=-4.3 (SD=2.0), *t*=-1.8, *p*=.088, *d*=0.8 (large), no significant effect |
| P3 standard amplitude (µV) | M=0.1 (SD=1.4) | M=0.7 (SD=1.2), *t*=-1.3, *p*= .215, *d*=0.5, (moderate), no significant effect | M=0.1 (SD=2.1), *t*=-0.0, *p*=.963, *d*=0.0 (small), no significant effect | M=1.6 (SD=1.8), *t*=-2.3, *p*=.025*, *d*=1.0 (large), Mixed delirium significantly higher |
| P3 deviant amplitude (µV) | M=-0.1 (SD=2.3), | M=0.6 (SD=0.9), *W*=-1.5, *p*= .146, *d*=0.4, (small to moderate), no significant effect | M=-0.5 (SD=3.5), *t*=0.4, *p*=.717, *d*=0.2 (small), no significant effect | M=2.1 (SD=2.6), *t*=-2.1, *p*=.041*, *d*=0.9 (large), Mixed delirium significantly higher |
| MMN amplitude (µV) | M=-0.8 (SD=1.2), | M=-1.5 (SD=1.3), *t*=1.5, *p*= .142, *d*=0.6, (moderate to large), no significant effect | M=-1.6 (SD=2.0), *t*=1.2, *p*=.245, *d*=0.6 (moderate), no significant effect | M=-0.6 (SD=0.7), *t*=-0.4, *p*=.716, *d*=0.2 (small), no significant effect |
| Note. *t*=independent samples t-test statistic; *W*=Welch’s t-test statistic; *d*= Cohens *d*; MMN=Mismatch negativity; μV= microvolts; * indicate statistical significance. | | | | |


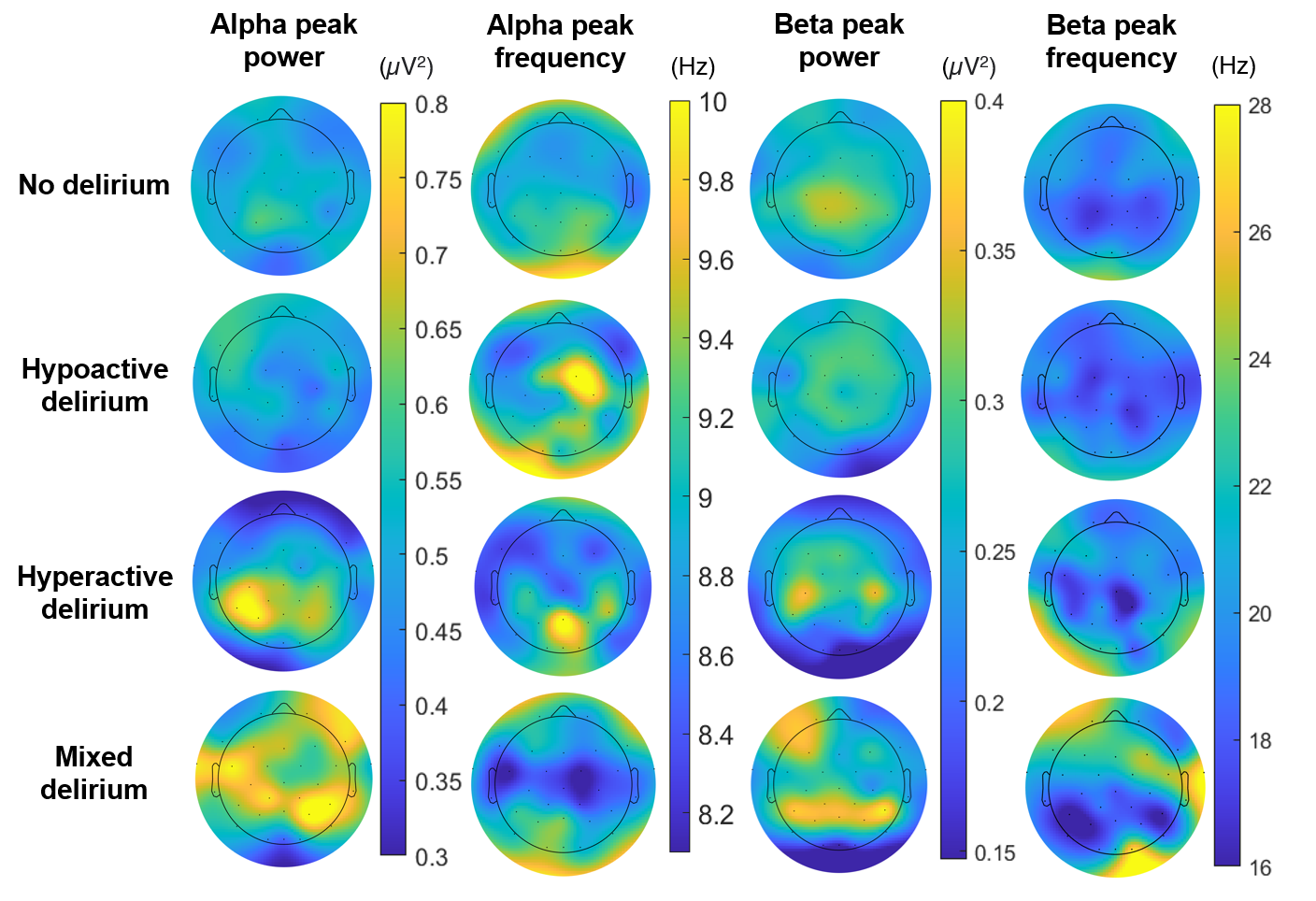


**Supplementary Figure 2.** Group averaged topographic plots show alpha and beta peak power (μV^2^) and peak frequency (Hz) during eyes open resting state recording for delirium subtype (hypoactive n=10; hyperactive n=5; mixed n=6) and no delirium (n=37) groups. μV^2^= microvolts squared; Hz= hertz.


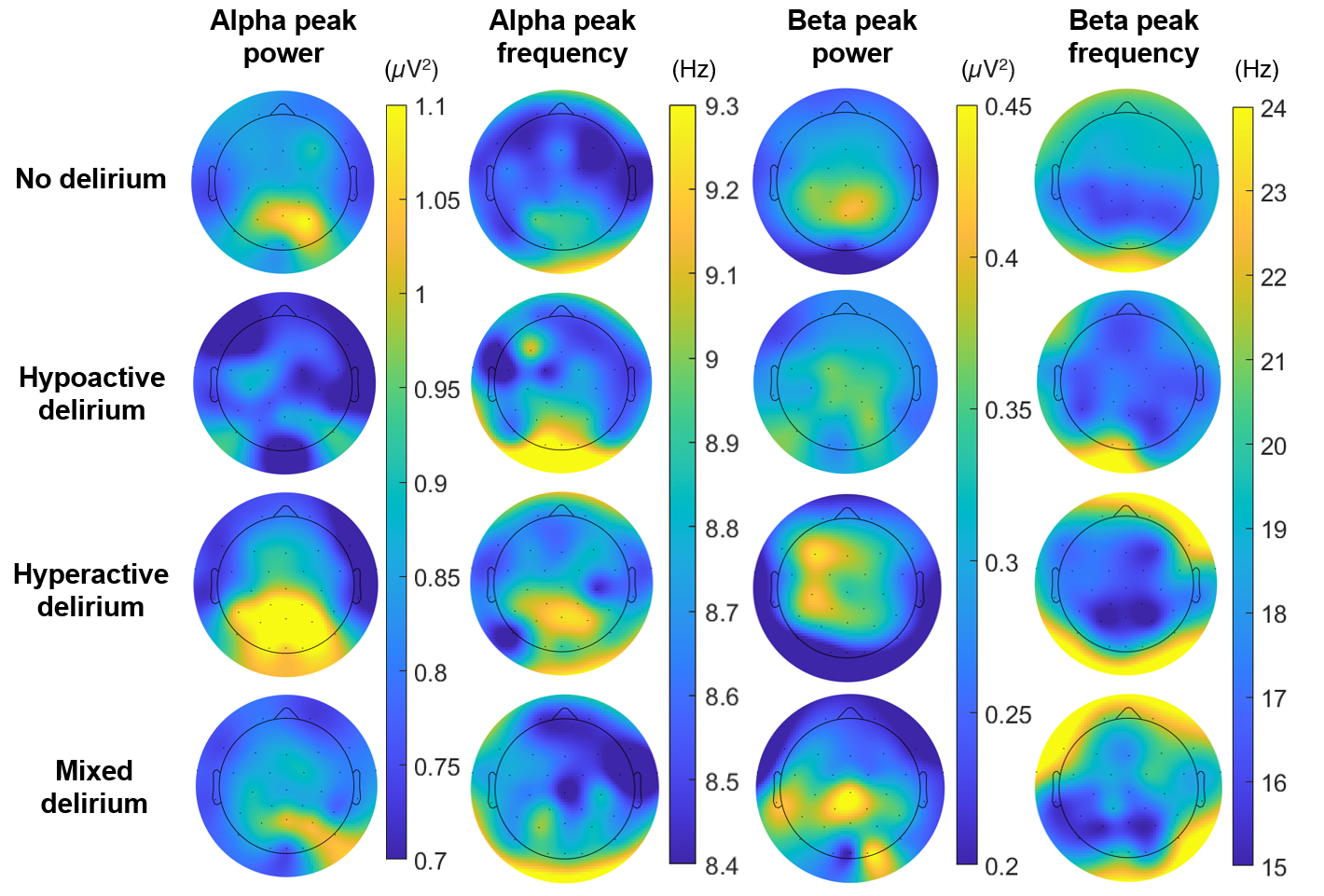


**Supplementary Figure 3.** Group averaged topographic plots show alpha and beta peak power (μV^2^) and peak frequency (Hz) during eyes closed resting state recording for delirium subtype (hypoactive n=10; hyperactive n=5; mixed n=6) and no delirium (n=37) groups. μV^2^= microvolts squared; Hz= hertz.

**Supplementary Table 8.** Individual data points for eyes open and closed periodic and aperiodic components in those who developed hyperactive (n=5) and mixed delirium (n=6).

|  | Alpha BW  (μV^2^) | | Alpha PP  (μV^2^) | | Alpha PF  (μV^2^) | | Beta BW  (μV^2^) | | Beta PP  (μV^2^) | | Beta PF  (μV^2^) | | Offset  (μV^2^) | | Exponent (μV^2^/Hz) | |
| --- | --- | --- | --- | --- | --- | --- | --- | --- | --- | --- | --- | --- | --- | --- | --- | --- |
|  | EO | EC | EO | EC | EO | EC | EO | EC | EO | EC | EO | EC | EO | EC | EO | EC |
| **Hyperactive** |  |  |  |  |  |  |  |  |  |  |  |  |  |  |  |  |
| Hyperactive 1 | 2.79 | 2.15 | 1.08 | 1.74 | 9.19 | 8.53 | 6.07 | 5.95 | 0.23 | 0.38 | 16.22 | 15.96 | -0.43 | -0.12 | 0.65 | 0.85 |
| Hyperactive 2 | 1.11 | 1.29 | 0.13 | 0.47 | 9.82 | 8.90 | 2.45 | 3.09 | 0.15 | 0.18 | 25.39 | 15.94 | 0.08 | 0.20 | 0.88 | 0.90 |
| Hyperactive 3 | 2.68 | 2.98 | 0.25 | 0.76 | 10.55 | 9.95 | 4.13 | 5.21 | 0.24 | 0.33 | 22.35 | 21.75 | -0.38 | -0.50 | 0.47 | 0.38 |
| Hyperactive 4 | 1.86 | 2.60 | 0.55 | 1.60 | 9.10 | 9.65 | 5.38 | 4.52 | 0.48 | 0.55 | 16.43 | 14.34 | -0.13 | 0.09 | 0.76 | 0.90 |
| Hyperactive 5 | 2.90 | 2.70 | 0.89 | 1.07 | 8.07 | 8.34 | 3.93 | 5.00 | 0.23 | 0.25 | 14.91 | 15.60 | 0.46 | 0.53 | 1.48 | 1.52 |
| **Mixed** |  |  |  |  |  |  |  |  |  |  |  |  |  |  |  |  |
| Mixed 1 | 3.29 | 2.40 | 0.84 | 1.33 | 10.72 | 8.89 | 5.54 | 5.16 | 0.41 | 0.52 | 18.97 | 17.68 | -0.27 | -0.20 | 0.89 | 0.85 |
| Mixed 2 | 1.83 | 2.25 | 0.42 | 0.51 | 7.58 | 7.60 | 2.92 | 2.00 | 0.25 | 0.29 | 17.69 | 15.87 | -0.46 | -0.47 | 0.77 | 0.79 |
| Mixed 3 | 1.18 | 2.11 | 0.14 | 0.56 | 11.19 | 9.96 | 3.75 | 4.40 | 0.24 | 0.20 | 19.67 | 18.11 | -0.20 | -0.07 | 1.09 | 1.14 |
| Mixed 4 | 2.00 | 2.26 | 0.85 | 1.21 | 6.70 | 6.54 | 3.45 | 1.99 | 0.26 | 0.33 | 18.33 | 17.06 | 0.01 | 0.27 | 1.16 | 1.57 |
| Mixed 5 | 2.82 | 2.37 | 0.74 | 1.19 | 9.60 | 8.73 | 3.84 | 6.13 | 0.44 | 0.41 | 17.41 | 16.53 | -0.44 | -0.18 | 0.52 | 0.76 |
| Mixed 6 | 1.75 | 2.68 | 0.08 | 0.65 | 9.73 | 11.31 | 5.85 | 2.63 | 0.21 | 0.17 | 25.57 | 22.03 | -0.35 | -0.59 | 0.88 | 0.69 |
| Note. BW= bandwidth; PP= peak power; PF= peak frequency; EO= eyes open; EC= eyes closed; μV^2^= microvolts squared; Hz= hertz. | | | | | | | | | | | | | | | | |
